# New Appliances and Things Medical

**Published:** 1901-05-18

**Authors:** 


### NEW APPLIANCES AND THINGS MEDICAL.

[We shall be glad to receive, at our Office, 28 & 29 Southampton Street, Strand, London, W.C., from the manufacturers, specimens of all new preparations and appliances which may be brought out from time to time.]

### CYPRIDOL.

(Vial, 8 Rue Vivienne, Paris. London Agents, Wilcox, Jozeau & Co., 49 Haymarket, London, S.W.)

Cypridol is a new oleacious preparation of mercuric iodide. The preparation is carefully standardized, and is of 1 per cent. strength. By a special process the vehicle, sterilised oil, is enabled to take up this comparatively large quantity of the iodide, which, as is well known, is by ordinary processes very refractive to solution in oils. The advantages of exhibiting this mercuric preparation dissolved in oil are very considerable, since whether administered by the mouth or by hypodermic injection, the irritating properties of the drug are reduced to a minimum, thus enabling the physician to pursue a course of mercurial treatment for a considerable period of time without introducing the complications of gastric or intestinal irritation. The drug is dispensed in capsules, each of which contains $\frac{1}{32}$ grain of mercuric iodide. For hypodermic administration 8 to 16 minims of the 1 per cent. solution should be injected into the muscular tissues daily.

### SOOTENE.

(The Unique Trading Company, Rotherham, Yorks.)

Sootene is a new dry powder fire-extinguisher; it is contained in long cylinders, with a lid which can easily be removed in cases of emergency. The contained powder must be forcibly thrown on to the flames, and the carbonic acid gas thereby liberated is capable of extinguishing in a marvellously short space of time very alarming conflagrations. The advantages of such a simple means of controlling what might otherwise be a most dangerous fire are self-evident to the meanest intelligence, and should be of the greatest use in private houses where more elaborate fire-extinguishing means cannot be provided. Unlike water, the powder cannot do any damage to furniture or other property; it cannot evaporate, and will keep indefinitely, and is ever ready at hand and capable of being used by the most inexperienced person.

